# Perioperative Intravenous Lidocaine and Early Biochemical Outcomes After Robotic-Assisted Radical Prostatectomy: A Clinical Study Within the Framework of Perioperative Metabolic-Inflammatory Modulation

**DOI:** 10.3390/metabo16030209

**Published:** 2026-03-20

**Authors:** Georgiana Maria Popa, Simona-Alina Abu-Awwad, Ahmed Abu-Awwad, Nicolae Ovidiu Pop, Parascovia Pop, Carmen Ioana Marta, Anca Mihaela Bina, Erika Bimbo Szuhai, Adriana Cacuci, Adrian Gheorghe Osiceanu, Ciprian Dumitru Puscas, Teodor Traian Maghiar, Mihai Octavian Botea

**Affiliations:** 1 Pelican Hospital, Corneliu Coposu Street 2, 410450 Oradea, Romania; pascalau.georgianamaria@student.uoradea.ro (G.M.P.); adrianacacuci@yahoo.com (A.C.); cipripuski1980@gmail.com (C.D.P.); mbotea@uoradea.ro (M.O.B.); 2 Department of Surgical Disciplines, Faculty of Medicine and Pharmacy, University of Oradea, 1st December Square 10, 410073 Oradea, Romania; pop.nicolaeovidiu@didactic.uoradea.ro (N.O.P.); parascovia.pop@didactic.uoradea.ro (P.P.); 3 Doctoral School of Biomedical Sciences, University of Oradea, 410087 Oradea, Romania; 4 Department of Obstetrics and Gynecology, “Victor Babes” University of Medicine and Pharmacy Timisoara, Eftimie Murgu Square 2, 300041 Timisoara, Romania; alina.abuawwad@umft.ro (S.-A.A.-A.); carmen.marta@umft.ro (C.I.M.); lungu.anca@umft.ro (A.M.B.); 5 Clinic of Obstetrics and Gynecology, “Pius Brinzeu” County Clinical Emergency Hospital, 300723 Timisoara, Romania; 6 Department XV—Discipline of Orthopedics—Traumatology, “Victor Babes” University of Medicine and Pharmacy, Eftimie Murgu Square 2, 300041 Timisoara, Romania; ahm.abuawwad@umft.ro; 7 Clinic of Orthopedics, “Pius Brinzeu” County Clinical Emergency Hospital, 300723 Timisoara, Romania; 8 Department of Morphological Disciplines, Faculty of Medicine and Pharmacy, University of Oradea, 1st December Square 10, 410073 Oradea, Romania; osiceanuadrian@yahoo.com (A.G.O.); uro_doruletul@yahoo.com (T.T.M.)

**Keywords:** prostate cancer, robotic-assisted radical prostatectomy, perioperative metabolic–inflammatory modulation, immunometabolism, intravenous lidocaine, PSA dynamics, surgical stress response, inflammatory–metabolic crosstalk, IL-6, lactate

## Abstract

Background: The perioperative period in cancer surgery is characterized by transient metabolic and inflammatory perturbations that may influence early postoperative biochemical dynamics. Surgical stress induces insulin resistance, hyperglycemia, cytokine activation, and metabolic shifts that interact with tumor cell signaling pathways. Intravenous lidocaine has been associated with anti-inflammatory and systemic stabilizing effects beyond analgesia. We investigated whether perioperative lidocaine administration during robotic-assisted radical prostatectomy (RARP) is associated with early postoperative prostate-specific antigen (PSA) dynamics within the context of perioperative metabolic–inflammatory modulation. Methods: In this single-center retrospective cohort study, 180 patients undergoing RARP for localized or locally advanced prostate cancer were stratified according to perioperative intravenous lidocaine exposure. The primary endpoint was undetectable PSA (<0.1 ng/mL) at 6–12 weeks postoperatively. Secondary endpoints included PSA detectability at 3 and 6 months and time to first detectable PSA. Multivariable logistic and Cox regression models were adjusted for established oncologic risk factors. Perioperative glycemic variation, intraoperative lactate dynamics, and postoperative IL-6 levels were analyzed as indicators of stress-induced metabolic activation. Results: Lidocaine exposure was independently associated with higher odds of undetectable PSA at 6–12 weeks (OR 2.10, 95% CI 1.15–3.85) and at subsequent time points. In Cox analysis, lidocaine was associated with a reduced hazard of PSA detectability (HR 0.58, 95% CI 0.37–0.92). Patients receiving lidocaine demonstrated significantly attenuated perioperative hyperglycemia, lower lactate elevation, and reduced IL-6 response. Conclusions: Perioperative intravenous lidocaine administration during RARP was associated with more favorable early PSA dynamics and attenuation of perioperative metabolic–inflammatory activation. Given the retrospective and non-randomized design of the study, these findings should be interpreted as associative and hypothesis-generating, and warrant confirmation in prospective controlled investigations.

## 1. Introduction

Prostate cancer continues to represent one of the most prevalent malignancies in men worldwide, and robotic-assisted radical prostatectomy (RARP) has become a highly standardized and widely adopted surgical approach for patients with localized and selected locally advanced disease. From a technical perspective, modern robotic surgery offers remarkable precision and reproducibility. Yet, even in this context of procedural uniformity, early postoperative biochemical outcomes are not always identical among patients with apparently comparable pathological characteristics [1,2].

After radical prostatectomy, prostate-specific antigen (PSA) levels are expected to decline to undetectable values within the first postoperative weeks. When PSA remains detectable early after surgery, this finding is typically attributed to established tumor-related determinants such as pathological stage, grade group, margin status, or lymph node involvement. These factors undoubtedly remain central to oncologic risk stratification [1,3]. However, surgery itself is not a biologically neutral event. It triggers a systemic stress response characterized by sympathetic activation, endocrine fluctuations, inflammatory mediator release, and measurable shifts in immune and metabolic homeostasis. In this sense, the perioperative period represents a transient but biologically active phase that may influence tumor-host interactions beyond the purely anatomical extent of resection [4,5].

The present study focuses on early postoperative PSA detectability rather than biochemical recurrence, which represents a later oncologic endpoint.

Increasing attention has been directed toward the concept that perioperative systemic conditions can modulate early cancer-related processes. Surgical trauma induces a coordinated metabolic and inflammatory response that may transiently influence tumor–host interactions. Within this framework, the interplay between immune regulation and cellular metabolism, often referred to as immunometabolism, has become an important theme in understanding cancer development and progression [4,6,7].

These systemic responses are also accompanied by transient metabolic alterations characterized by stress-related insulin resistance and perioperative hyperglycemia [4,8]. Therefore, perioperative metabolic perturbations may theoretically influence early postoperative PSA dynamics not only as a reflection of residual tumor burden, but also as a consequence of transient systemic metabolic modulation.

Intravenous lidocaine is commonly incorporated into perioperative analgesic strategies due to its opioid-sparing and anti-hyperalgesic effects [9,10]. Beyond analgesia, however, lidocaine has been shown to exert anti-inflammatory and immunomodulatory actions, including attenuation of cytokine release and modulation of leukocyte activation [11,12]. Experimental data also suggest that local anesthetics may interfere with cellular signaling pathways implicated in tumor cell migration and invasion in preclinical models [13,14]. While these mechanistic observations should be interpreted cautiously in clinical settings, they raise a clinically meaningful question: could perioperative systemic modulation influence early oncologic biomarkers?

In prostate cancer surgery, early PSA dynamics provide a standardized and highly sensitive indicator of postoperative biochemical status [1,15]. Although PSA suppression primarily reflects successful tumor removal, it may also be shaped by systemic conditions influencing residual tumor cell activity in the immediate postoperative phase. Exploring this possibility does not diminish the importance of classical pathological predictors; rather, it acknowledges that tumor biology unfolds within a host environment that is temporarily altered by surgical and anesthetic factors [16,17].

Previous clinical investigations evaluating perioperative intravenous lidocaine in prostate surgery have primarily focused on analgesic, inflammatory, or recovery-related outcomes rather than oncologic biomarkers. For instance, a recent randomized study in patients undergoing robotic-assisted radical prostatectomy demonstrated that perioperative lidocaine administration modified inflammatory markers such as myeloperoxidase and neutrophil elastase, although no significant effect on biochemical recurrence was observed [18]. Consequently, whether perioperative lidocaine exposure may be associated with early postoperative PSA dynamics, a sensitive indicator of early biochemical status following prostatectomy, remains insufficiently explored.

The present study was therefore designed to address this gap by examining the association between perioperative lidocaine administration and early PSA detectability, while simultaneously integrating perioperative metabolic and inflammatory markers within a unified analytical framework.

In this context, we evaluated whether perioperative intravenous lidocaine administration during RARP is associated with early postoperative PSA detectability, considered within the broader context of perioperative metabolic-inflammatory modulation. By integrating clinical, pathological, inflammatory, and biochemical data and adjusting for established oncologic risk factors, we sought to determine whether systemic perioperative influences may contribute to early biochemical outcomes after prostatectomy. Our objective was not to advance a definitive mechanistic conclusion, but to investigate whether the perioperative metabolic window represents a clinically relevant dimension in prostate cancer management [14,19].

## 2. Materials and Methods

### 2.1. Study Design and Ethical Approval

We conducted a single-center retrospective cohort study at Pelican Clinical Hospital, Oradea, Romania, a tertiary referral institution with an established robotic urologic surgery program. The aim of the study was to evaluate the association between perioperative intravenous lidocaine administration and early postoperative PSA dynamics, within the framework of perioperative metabolic-inflammatory modulation in prostate cancer surgery.

The index date for all analyses was defined as the date of surgery. Clinical, perioperative, pathological, and follow-up data were extracted from the institutional electronic medical record system using a predefined standardized data collection template. To ensure data accuracy, records were independently reviewed by two investigators, and discrepancies were resolved by consensus.

The study protocol was approved by the Institutional Ethics Committee of Pelican Clinical Hospital (approval No. 2166/19 October 2021). All procedures were conducted in accordance with the Declaration of Helsinki [20]. All patients had provided written informed consent for surgical treatment and for the anonymized use of their clinical data for research purposes.

### 2.2. Study Population

Between the predefined study dates, 180 consecutive patients undergoing elective robotic-assisted radical prostatectomy for histologically confirmed prostate adenocarcinoma were included in the final analysis.

Eligible patients met the following inclusion criteria:

- Age between 40 and 80 years,
- Clinically localized or locally advanced disease (cT1–cT3),
- Absence of distant metastases (M0) based on guideline-based preoperative staging [21],
- Available preoperative PSA measurement,
- At least one postoperative PSA assessment within the predefined early follow-up interval.

To reduce potential confounding related to prior oncologic interventions, patients who had received neoadjuvant androgen deprivation therapy, chemotherapy, or pelvic radiotherapy were excluded. Additional exclusion criteria included metastatic disease at diagnosis, intraoperative conversion to open radical prostatectomy, major intraoperative complications that significantly altered the anesthetic plan, known hypersensitivity to lidocaine, severe hepatic or renal dysfunction, clinically significant cardiac disease affecting lidocaine metabolism, chronic opioid therapy, long-term corticosteroid use, active inflammatory or autoimmune disorders, or other active malignancies requiring systemic treatment.

Patients were stratified into two groups according to the documented intraoperative anesthetic management: Lidocaine Group (LG): patients who received perioperative intravenous lidocaine infusion; and Control Group (CG): patients managed with the same standardized anesthetic protocol without lidocaine administration.

The decision to administer lidocaine was based on the institutional anesthetic protocol in use during the study period and the attending anesthesiologist’s perioperative strategy, independent of tumor characteristics or expected oncologic outcomes.

### 2.3. Surgical and Anesthetic Management

All procedures were performed using the da Vinci robotic platform by the same experienced surgical team, minimizing inter-operator variability. A standardized transperitoneal RARP technique was applied in all cases. Pelvic lymph node dissection was performed according to preoperative risk stratification and intraoperative assessment [22].

General anesthesia was induced using intravenous propofol, fentanyl, and rocuronium. Maintenance was achieved with sevoflurane in an oxygen-air mixture, titrated according to hemodynamic responses and institutional practice. Intraoperative opioid administration was guided by clinical parameters and anesthetic depth monitoring.

Perioperative fluid therapy, antibiotic prophylaxis, and thromboprophylaxis were administered according to institutional protocols and were applied uniformly to both groups. Postoperative analgesia consisted of a multimodal regimen including non-opioid analgesics, with rescue opioids administered as clinically indicated.

Total perioperative opioid consumption was recorded and converted to morphine milligram equivalents (MME) using standardized conversion factors. Rescue opioid administration was defined as any additional opioid requirement beyond the standard multimodal analgesic regimen during the first 24 h postoperatively.

Perioperative corticosteroids were not administered as part of the institutional anesthetic protocol.

### 2.4. Lidocaine Administration Protocol

In the Lidocaine Group, intravenous lidocaine was administered immediately after induction of anesthesia as a bolus of 1.5 mg/kg (based on actual body weight), followed by a continuous infusion at 1.5 mg/kg/h throughout the surgical procedure. The total dose did not exceed 300 mg per patient. The infusion was discontinued at the completion of surgery and was not continued postoperatively.

All patients were continuously monitored intraoperatively according to standard anesthetic practice, including electrocardiography, invasive blood pressure monitoring when indicated, pulse oximetry, capnography, and temperature monitoring. No cases of clinically suspected local anesthetic systemic toxicity were documented.

Patients in the Control Group underwent identical surgical and anesthetic management, with the sole difference being the omission of systemic lidocaine administration.

### 2.5. Data Collection and Variables

Demographic variables included age and body mass index (BMI). Clinical variables comprised ASA physical status classification and relevant comorbidities.

Perioperative variables included: duration of surgery, duration of anesthesia, pneumoperitoneum pressure, time spent in Trendelenburg position, estimated blood loss, intraoperative fluid administration, urine output, need for blood transfusion.

Intraoperative hemodynamic parameters were recorded at predefined time points: T0: baseline before induction, T1: after induction, T2: after pneumoperitoneum, T3: after Trendelenburg positioning, T4: 60 min intraoperatively, T5: end of surgery.

Episodes of hypotension (MAP < 65 mmHg), hypertension (SBP > 160 mmHg), vasopressor use, and number of vasopressor boluses were documented.

Respiratory parameters included end-tidal CO_2_ (EtCO_2_), arterial PaCO_2_, peak airway pressure, plateau pressure, dynamic compliance, minute ventilation, and peripheral oxygen saturation (SpO_2_).

Oncologic variables comprised: preoperative PSA, biopsy ISUP grade group, pathological tumor stage (pT), lymph node status (pN), surgical margin status and final ISUP grade group.

All pathological assessments were performed according to standard institutional protocols consistent with international guidelines.

Inflammatory markers (leukocyte count, neutrophil-to-lymphocyte ratio (NRL) [23], and CRP) were considered surrogate indicators of perioperative metabolic-inflammatory activation, given the well-established interplay between systemic inflammation, immune metabolism, and tumor microenvironment dynamics.

In addition to standard inflammatory markers, perioperative metabolic parameters were systematically recorded to further characterize the systemic stress response. Blood glucose levels were collected at three predefined time points: pre-induction (T0), intraoperatively at 60 min after pneumoperitoneum (T4), and at 24 h postoperatively. Perioperative glycemic variation (Δ glucose) was calculated as the difference between intraoperative and baseline values.

Arterial lactate levels were extracted from routine intraoperative blood gas analyses at baseline and during peak surgical stress (T3–T4). Lactate variation (Δ lactate) was calculated to quantify stress-induced metabolic activation.

Serum interleukin-6 (IL-6) concentrations were measured preoperatively and at 6–24 h postoperatively, reflecting early inflammatory-metabolic activation [12]. Δ IL-6 was calculated to assess the magnitude of the perioperative cytokine response. These parameters were analyzed as surrogate indicators of systemic metabolic-inflammatory modulation. IL-6 measurements were obtained as part of the routine perioperative laboratory protocol during the study period and were available for the entire study cohort. No selective inclusion based on biomarker availability was applied.

### 2.6. PSA Assessment and Outcome Definitions

Serum PSA measurements were performed in the same certified institutional laboratory using a standardized electrochemiluminescence immunoassay throughout the study period. No changes in assay methodology occurred during the study interval.

The first postoperative PSA measurement was obtained within a clinically predefined window of 6 to 12 weeks after surgery, reflecting routine follow-up practice. Because this interval partially overlaps with the approximate 3-month follow-up assessment, the temporal sequence of the early PSA endpoints should be interpreted pragmatically rather than as strictly independent time points. The 6–12-week assessment was selected as the primary endpoint to capture the earliest clinically relevant biochemical status after surgery, whereas the 3- and 6-month measurements were analyzed as subsequent confirmatory follow-up assessments.

The first postoperative PSA measurement was defined as the value obtained between 6 and 12 weeks after surgery. Subsequent PSA assessments at approximately 3 and 6 months were recorded according to routine follow-up practice.

An undetectable PSA was defined as <0.1 ng/mL [24], in accordance with the institutional laboratory threshold. PSA persistence was defined as a value ≥ 0.1 ng/mL at the first postoperative assessment.

The primary endpoint was the proportion of patients with undetectable PSA at 6–12 weeks postoperatively. Secondary endpoints included PSA detectability at 3 and 6 months, time to first detectable PSA (≥0.1 ng/mL), and early postoperative recovery outcomes.

Time to first detectable PSA was estimated from the date of surgery to the first recorded PSA value ≥ 0.1 ng/mL during scheduled follow-up assessments. Patients without detectable PSA during follow-up were censored at the date of their last available PSA measurement.

Early postoperative PSA dynamics were considered a clinically relevant biochemical indicator following prostatectomy, reflecting the degree of PSA suppression after prostate removal and potentially influenced by both pathological tumor characteristics and perioperative systemic conditions.

It is important to distinguish between three related but conceptually different postoperative PSA phenomena: early detectable PSA, PSA persistence, and biochemical recurrence. Early detectable PSA refers to the presence of measurable PSA during the first postoperative assessment period and may reflect delayed PSA clearance, assay sensitivity, or residual benign or malignant prostatic tissue. PSA persistence is typically defined as PSA levels remaining ≥ 0.1 ng/mL within the early postoperative period (usually within 6–12 weeks after surgery) and suggests incomplete PSA suppression after prostate removal. In contrast, biochemical recurrence (BCR) represents a later oncologic endpoint, generally defined as a confirmed PSA rise ≥ 0.2 ng/mL after an initially undetectable postoperative value. Because our study focuses on early postoperative PSA detectability within the first months after surgery, the analyses presented here relate primarily to early biochemical dynamics rather than to established biochemical recurrence.

### 2.7. Statistical Analysis

Statistical analyses were performed using GraphPad Prism version 10 (GraphPad Software, San Diego, CA, USA) and MedCalc Statistical Software version 23.0 (MedCalc Software Ltd., Ostend, Belgium).

Continuous variables were tested for normality using the Shapiro–Wilk test. Normally distributed variables are presented as mean ± standard deviation and were compared using the independent samples *t*-test. Non-normally distributed data are presented as median (interquartile range) and were compared using the Mann–Whitney U test. Categorical variables are reported as absolute numbers and percentages and were compared using the Chi-square or Fisher’s exact test, as appropriate.

Multivariable logistic regression models were constructed to identify independent predictors of undetectable PSA at 6–12 weeks, 3 months, and 6 months. Covariates were selected a priori based on established clinical relevance and included perioperative intravenous lidocaine administration, preoperative PSA, pathological stage, final ISUP grade group, and surgical margin status.

Time to first detectable PSA was analyzed using Kaplan–Meier survival curves and compared with the log-rank test. A multivariable Cox proportional hazards regression model was constructed to explore predictors of early postoperative PSA detectability. Given that PSA measurements were obtained at predefined follow-up visits rather than through continuous monitoring, the exact timing of PSA detectability is interval-censored between assessments. Therefore, the time-to-event analysis should be interpreted as exploratory. The proportional hazards assumption was assessed using standard residual-based diagnostics.

Inflammatory variables were additionally analyzed to explore patterns consistent with differential perioperative metabolic activation between groups.

To further explore the potential contribution of perioperative metabolic activation to early biochemical outcomes, a sensitivity multivariable logistic regression model was constructed including Δ IL-6 and Δ lactate in addition to the predefined oncologic covariates. Continuous metabolic variables were entered using clinically interpretable scaling. This exploratory model was designed to assess whether the inclusion of metabolic-inflammatory parameters influenced the magnitude of the association between lidocaine exposure and early PSA dynamics.

All statistical tests were two-sided, and a *p*-value < 0.05 was considered statistically significant.

## 3. Results

A total of 180 patients were included in the analysis, 90 in the Lidocaine Group (LG) and 90 in the Control Group (CG). The two groups were highly comparable at baseline, without statistically significant differences in demographic, clinical, or pathological parameters (Table 1). Baseline inflammatory parameters, reflecting systemic metabolic–inflammatory status, were comparable between groups.

Mean age and BMI were similar between groups, and the distribution of ASA physical status reflected a comparable preoperative anesthetic risk profile. Preoperative PSA levels, inflammatory markers (leukocyte count, NLR, CRP), and tumor characteristics did not differ significantly. Likewise, clinical stage, biopsy ISUP grade group, pathological stage, and final ISUP grade distribution were evenly balanced. The rates of positive surgical margins and lymph node involvement were also comparable.

Importantly, operative time and estimated blood loss were similar between groups, suggesting that major baseline imbalances or procedural complexity were unlikely to fully explain the observed differences in outcomes, although residual confounding cannot be excluded.

Intraoperative variables were nearly identical between groups (Table 2). Duration of surgery and anesthesia did not differ significantly, and pneumoperitoneum pressure as well as time spent in Trendelenburg positioning were comparable.

Estimated blood loss, intraoperative fluid administration, urine output, and the need for blood transfusion were also similar. The proportion of patients undergoing RARP alone versus RARP combined with pelvic lymph node dissection was evenly distributed.

Clear differences emerged when analyzing intraoperative hemodynamic parameters (Table 3). While baseline mean arterial pressure (MAP) and heart rate were comparable, patients receiving intravenous lidocaine showed more stable intraoperative hemodynamic profiles during the critical phases of surgery.

Following induction, pneumoperitoneum, and Trendelenburg positioning (T1–T4), MAP values were more stable in the Lidocaine Group, with statistically significant differences at several time points. Similarly, heart rate remained consistently lower and more controlled in the lidocaine cohort during intraoperative stress periods.

From a clinical standpoint, these differences translated into fewer hypotensive and hypertensive episodes in the Lidocaine Group. Patients receiving lidocaine experienced significantly fewer episodes of MAP < 65 mmHg, shorter durations of hypotension, and fewer hypertensive events. Moreover, vasopressor requirements were lower both in terms of proportion of patients requiring support and the number of boluses administered.

Despite the differences observed in hemodynamic variables, ventilatory and respiratory parameters remained comparable between groups (Table 4).

End-tidal CO_2_ and arterial PaCO_2_ followed the expected intraoperative pattern, increasing after pneumoperitoneum and Trendelenburg positioning, but without significant intergroup differences at any time point. Peak airway pressure and plateau pressure increased during Trendelenburg, as anticipated, yet remained similar between groups. Dynamic compliance, minute ventilation, and peripheral oxygen saturation also showed no statistically significant differences.

These findings may be consistent with a pattern compatible with attenuated neuroendocrine stress activation, although causal inference cannot be established in this observational setting.

Perioperative metabolic parameters revealed a pattern consistent with attenuated stress-induced metabolic activation in the Lidocaine Group. Baseline blood glucose and lactate levels were comparable between groups. However, intraoperative glucose levels were significantly lower in patients receiving lidocaine, with a reduced Δ glucose compared to controls. Similarly, peak intraoperative lactate levels and Δ lactate were significantly attenuated in the lidocaine cohort.

IL-6 values were available for all included patients and were analyzed according to the predefined perioperative sampling protocol. Postoperative IL-6 concentrations demonstrated a significantly smaller increase in the Lidocaine Group, despite comparable baseline values. The magnitude of Δ IL-6 was markedly reduced compared to controls, suggesting a pattern compatible with reduced early inflammatory-metabolic activation.

Together, these findings suggest a pattern compatible with attenuated perioperative metabolic and cytokine perturbations in patients who received intravenous lidocaine.

Postoperative recovery parameters showed several clinically meaningful differences (Table 5). Time to extubation was significantly shorter in the Lidocaine Group, suggesting faster emergence from anesthesia.

Although rates of postoperative nausea and vomiting (PONV) and postoperative ileus were lower in the lidocaine cohort, these differences did not reach statistical significance. However, length of hospital stay was significantly reduced in patients receiving lidocaine, which may reflect a more favorable overall recovery profile.

Rates of overall complications (Clavien-Dindo ≥ II), as well as stratified complication grades and 30-day readmission rates, were numerically lower in the Lidocaine Group but did not reach statistical significance. Nevertheless, the consistent direction of effect across several outcomes suggests a potential clinical benefit.

The shorter time to extubation and reduced length of hospital stay observed in the Lidocaine Group may further suggest a more favorable systemic recovery profile, potentially linked to attenuated perioperative metabolic stress.

Given the conceptual framework of perioperative metabolic-inflammatory modulation, multivariable models were constructed to explore whether lidocaine exposure remained independently associated with early biochemical outcomes.

Multivariable logistic regression analysis demonstrated a consistent and independent association between perioperative intravenous lidocaine and undetectable PSA (<0.1 ng/mL) at all evaluated postoperative time points (Table 6).

At 6–12 weeks, 3 months, and 6 months, lidocaine administration was associated with approximately a twofold higher likelihood of achieving undetectable PSA, even after adjusting for preoperative PSA, pathological stage, final ISUP grade group, and positive surgical margins.

This association remained independent of classical pathological predictors, suggesting that perioperative systemic factors beyond intrinsic tumor characteristics may be associated with early biochemical outcomes.

As expected, higher preoperative PSA, pathological stage pT3, higher ISUP grade (3–5), and positive surgical margins were independently associated with lower odds of undetectable PSA.

In a sensitivity multivariable model including Δ IL-6 and Δ lactate, the association between perioperative intravenous lidocaine and undetectable PSA at 6–12 weeks remained statistically significant, although the magnitude of the effect was partially attenuated compared with the primary model (Table 7). Δ IL-6 demonstrated an independent association with early PSA detectability, suggesting that greater perioperative inflammatory-metabolic activation was associated with a lower probability of achieving undetectable PSA. Δ lactate showed a consistent direction of effect, although statistical significance was borderline.

The inclusion of these metabolic parameters did not abolish the association between lidocaine exposure and early biochemical outcomes, but modestly reduced its effect size, supporting the hypothesis that perioperative metabolic modulation may be associated with early postoperative PSA dynamics.

Cox proportional hazards analysis provided an exploratory assessment of the temporal pattern of early PSA detectability. Perioperative intravenous lidocaine was associated with a reduced hazard of developing an early detectable postoperative PSA value during follow-up. (Table 8).

Conversely, higher preoperative PSA, pathological stage pT3, higher ISUP grade, and positive surgical margins were associated with increased risk of developing detectable postoperative PSA during follow-up.

Importantly, the protective association of lidocaine persisted after multivariable adjustment, suggesting a potential association with early biochemical dynamics following RARP.

Although differences did not reach statistical significance, a consistent directional trend toward lower inflammatory activation was observed in the Lidocaine Group. Key variables related to early PSA dynamics and perioperative lidocaine exposure are underlined to facilitate visual identification of the primary oncologic endpoints.

## 4. Discussion

In this retrospective cohort of patients undergoing robotic-assisted radical prostatectomy, perioperative intravenous lidocaine administration was independently associated with a higher likelihood of achieving undetectable PSA during early postoperative follow-up. This association was consistent at 6–12 weeks, 3 months, and 6 months, and remained significant after adjustment for established pathological determinants such as tumor stage, grade group, surgical margin status, and preoperative PSA [25]. In parallel, patients receiving lidocaine demonstrated greater intraoperative hemodynamic stability, reduced vasopressor requirements, faster postoperative recovery, and a coherent directional trend toward lower inflammatory activation.

It should be emphasized that early postoperative PSA dynamics represent a biochemical indicator following prostatectomy and should not be interpreted as a direct measure of residual tumor biological activity.

From an oncologic standpoint, early PSA suppression after radical prostatectomy is primarily interpreted as reflecting the completeness of prostate removal and the absence of detectable residual PSA-producing tissue [26]. In our cohort, classical predictors behaved exactly as expected: higher preoperative PSA, pathological stage pT3, higher ISUP grade group, and positive surgical margins [27] were all independently associated with reduced odds of early PSA negativity and increased hazard of developing detectable postoperative PSA. These findings reinforce the internal validity of the dataset and align with previous studies evaluating PSA persistence and early biochemical recurrence after prostatectomy.

What distinguishes our results is that the association between lidocaine exposure and early PSA suppression persisted independently of these tumor-related factors. This suggests that perioperative systemic conditions may contribute, at least in part, to early biochemical behavior. While the magnitude of effect does not overshadow intrinsic tumor biology, its consistency across multiple time points and analytical approaches supports the plausibility of a biologically plausible association.

Surgical trauma is accompanied by a coordinated stress response involving sympathetic activation, endocrine fluctuations, inflammatory mediator release, and measurable metabolic shifts [4,28]. Increasing evidence in oncology has emphasized the interplay between inflammation, immune regulation, and cellular metabolism, often conceptualized under the umbrella of immunometabolism [29]. The perioperative period therefore represents a transient yet biologically active phase during which tumor-host interactions may be influenced by systemic conditions beyond the local surgical field [28,30].

In addition to metabolic and inflammatory changes, surgical stress has been associated with transient alterations in antitumor immune surveillance. Experimental and clinical studies have reported perioperative suppression of natural killer (NK) cell activity and functional impairment of T-cell–mediated immune responses following major surgery. Because NK cells and cytotoxic T lymphocytes play a central role in controlling residual tumor cells and micrometastatic disease, perioperative immune suppression has been proposed as a potential mechanism linking surgical stress with early postoperative biochemical dynamics. Within this context, strategies that attenuate perioperative stress responses may theoretically contribute to preservation of perioperative immune competence [16,17].

Intravenous lidocaine, commonly integrated into enhanced recovery protocols for its analgesic and opioid-sparing properties, has been shown to exert anti-inflammatory and immunomodulatory effects [31]. Experimental data have demonstrated attenuation of pro-inflammatory cytokine release and modulation of leukocyte activity [32]. Preclinical studies further suggest that lidocaine may influence tumor biology through mechanisms including sodium-channel blockade, apoptosis induction, and reduced tumor cell migration and invasion [33]. Although translation of these mechanistic findings to clinical oncologic endpoints must be approached cautiously, they provide a conceptual framework within which our clinical observations can be interpreted.

An additional consideration relates to the potential biological implications of perioperative opioid exposure. Opioids have been reported to influence immune function and may modulate tumor-related signaling pathways through mechanisms including suppression of natural killer cell activity, promotion of angiogenic signaling, and modulation of inflammatory responses. Although clinical evidence remains heterogeneous, these observations have raised the hypothesis that opioid-sparing strategies could contribute to a more favorable perioperative host environment in cancer surgery. In this context, the opioid-sparing properties of intravenous lidocaine may represent an additional factor associated with perioperative systemic conditions that could influence early biological dynamics following tumor resection [30].

Intravenous lidocaine is increasingly incorporated into Enhanced Recovery After Surgery (ERAS) pathways due to its opioid-sparing properties, anti-inflammatory effects, and potential to accelerate postoperative recovery. ERAS protocols aim to attenuate the physiological stress response to surgery through multimodal strategies targeting analgesia, metabolic regulation, and early functional recovery. Within this framework, the metabolic and inflammatory attenuation observed in our cohort may be interpreted as consistent with the broader ERAS principle of perioperative stress modulation. Although our study was not designed to evaluate ERAS outcomes directly, the findings suggest that perioperative lidocaine may represent one component of integrated perioperative strategies aimed at optimizing systemic recovery during oncologic surgery [16,28].

In our study, routinely available inflammatory markers such as leukocyte count, neutrophil-to-lymphocyte ratio, and C-reactive protein did not demonstrate statistically significant differences between groups. Although a modest directional trend toward lower postoperative inflammatory activation was observed in patients receiving lidocaine, these findings should be interpreted cautiously, as the differences did not reach statistical significance.

In contrast, several perioperative metabolic and cytokine-related parameters demonstrated clearer between-group differences. Patients receiving lidocaine exhibited significantly attenuated perioperative glycemic elevation, lower intraoperative lactate increases, and a reduced postoperative IL-6 response. These findings suggest a pattern compatible with reduced perioperative metabolic-inflammatory activation in the lidocaine group. However, given the observational nature of the study, these observations should be interpreted as associative and exploratory rather than as evidence of a causal biological effect [34].

The observed intraoperative hemodynamic stability may further contribute to this interpretation. Reduced episodes of hypotension and hypertension, along with lower vasopressor requirements, may reflect blunted sympathetic and neuroendocrine activation [35]. Given that catecholamine release and stress hormone fluctuations are closely linked to metabolic dysregulation and immune modulation, improved intraoperative stability may translate into a more controlled systemic environment during a biologically sensitive period. From this perspective, any potential benefit associated with lidocaine may relate less to a direct antitumoral effect and more to its capacity to influence the perioperative host milieu during the perioperative metabolic window [36].

Surgical stress is also accompanied by transient insulin resistance and perioperative hyperglycemia, metabolic phenomena that may enhance glycolytic flux and cellular bioenergetic adaptation [4,8]. In addition, cancer metabolism itself is frequently characterized by increased aerobic glycolysis, commonly referred to as the Warburg effect, which supports rapid cellular proliferation and biosynthetic activity. In prostate cancer, androgen receptor (AR) signaling has been shown to regulate multiple metabolic pathways, including glucose utilization, lipid synthesis, and mitochondrial function, linking tumor growth to metabolic programming. Although these molecular mechanisms operate primarily at the tumor-cell level, they provide a conceptual framework suggesting that systemic metabolic conditions during the perioperative period may interact with tumor-related metabolic pathways [22,37].

Within this framework, perioperative metabolic perturbations such as transient hyperglycemia and increased lactate levels may theoretically interact with tumor-cell metabolic pathways. Surgical stress-related increases in circulating glucose and glycolytic flux may create a systemic environment that temporarily favors metabolic programs already utilized by tumor cells, providing a conceptual rationale for examining perioperative metabolic variables in relation to early oncologic biomarkers [7,19].

Sympathetic activation further amplifies β-adrenergic signaling pathways known to intersect with intracellular networks regulating proliferation and metabolic reprogramming [38]. In the present study, perioperative metabolic parameters, including glycemic variation, intraoperative lactate dynamics, and postoperative IL-6 elevation, demonstrated a coherent attenuation in patients receiving lidocaine. These findings suggest that perioperative metabolic–inflammatory modulation may extend beyond hemodynamic control to encompass modulation of stress-driven metabolic and inflammatory pathways. Transient perioperative hyperglycemia and lactate elevation reflect adaptive metabolic reprogramming under surgical stress, characterized by enhanced glycolytic flux and altered mitochondrial energetics [8]. It should be noted that perioperative lactate elevation in surgical settings is multifactorial and does not necessarily indicate tumor-related metabolic activity. Lactate dynamics may reflect a combination of transient tissue hypoperfusion, adrenergic stimulation–induced glycolysis, and anesthesia-related metabolic effects during surgical stress. Therefore, lactate should be interpreted primarily as a marker of systemic physiological stress rather than a tumor-specific metabolic signal [29]. The observed reduction in these metabolic shifts in the lidocaine group may reflect a pattern compatible with attenuation of sympathetic-driven bioenergetic activation. In parallel, the reduced IL-6 response supports modulation of inflammatory-metabolic crosstalk, a pathway increasingly implicated in tumor cell signaling and microenvironmental adaptation [39].

It should be acknowledged that perioperative IL-6 elevation is a well-recognized response to surgical trauma and represents a pleiotropic inflammatory mediator rather than a tumor-specific biomarker. Therefore, the observed IL-6 dynamics should primarily be interpreted as reflecting the magnitude of the surgical stress response rather than direct tumor-related biological processes [12,28].

While these observations do not establish causality, they provide a biologically plausible framework linking perioperative metabolic–inflammatory modulation to early biochemical dynamics following prostatectomy.

The sensitivity analysis incorporating Δ IL-6 and Δ lactate provides additional insight into the relationship between perioperative systemic modulation and early biochemical outcomes. The partial attenuation of the lidocaine effect after adjustment for these metabolic parameters suggests that stress-induced inflammatory and bioenergetic activation may represent one component of the observed association. Elevated perioperative IL-6 and lactate levels reflect coordinated inflammatory-metabolic responses to surgical stress, which have been implicated in tumor cell signaling, microenvironmental adaptation, and metabolic flexibility [39]. Although causality cannot be inferred, the persistence of the lidocaine association alongside a measurable reduction in metabolic perturbation supports a possible association between perioperative metabolic-inflammatory modulation and early postoperative PSA dynamics [18].

Clinical investigations examining anesthetic techniques and long-term oncologic outcomes have yielded heterogeneous results across tumor types [40,41]. Large, randomized trials in other malignancies have not consistently demonstrated survival differences attributable to anesthetic strategy [40]. However, such studies typically evaluate distant recurrence or survival endpoints, which are influenced by numerous variables over extended follow-up. Early postoperative biomarkers, such as PSA detectability, may represent a more immediate and sensitive indicator of perioperative biological influences.

In prostate cancer specifically, data linking anesthetic management to biochemical outcomes remain limited [40]. Most prior research has focused on inflammatory parameters or recovery-related endpoints rather than early PSA dynamics [41]. By integrating perioperative physiological data, inflammatory markers, and multivariable oncologic modeling within a single framework, our study contributes clinical evidence to a field that has largely been explored from experimental or theoretical perspectives.

It is important to emphasize that our findings do not challenge the central role of tumor biology in determining oncologic outcome. Pathological stage, grade group, and margin status remained dominant predictors of PSA behavior. Rather, our results suggest that perioperative systemic modulation may represent an additional layer of influence operating alongside intrinsic tumor characteristics. In this sense, the perioperative period may be viewed as a transient metabolic-inflammatory phase capable of shaping early tumor-related biomarkers.

While the retrospective design precludes definitive causal inference and mechanistic interpretations remain inferential, the internal coherence of the findings strengthens their credibility. Baseline characteristics were balanced, surgical parameters were comparable, and classical oncologic predictors demonstrated expected effects. The persistence of the association across logistic and Cox regression models provides exploratory support for the observed association within the limitations of an observational design.

These findings suggest that the perioperative environment may represent a biologically relevant context potentially influencing early postoperative biochemical markers. Even short-lived alterations in systemic metabolic and inflammatory regulation may influence early biochemical dynamics. Within the context of metabolic regulation in cancer development and progression, our study adds clinical evidence that perioperative modulation deserves further attention as a potentially relevant dimension of oncologic care.

### Strengths, Limitations and Future Directions

This study has several strengths that deserve emphasis. First, it was conducted in a relatively homogeneous cohort of patients undergoing robotic-assisted radical prostatectomy performed by the same experienced surgical team, which minimizes technical variability and enhances internal consistency. Surgical parameters, baseline demographic characteristics, and established pathological predictors were well balanced between groups, strengthening the interpretability of the comparative analyses. Second, we integrated perioperative physiological data, inflammatory markers, and multivariable oncologic modeling within a single analytical framework. This allowed us to explore the relationship between anesthetic management, systemic metabolic-inflammatory modulation, and early biochemical outcomes in a clinically coherent manner. Finally, the consistency of the association between lidocaine exposure and early PSA suppression across multiple time points and both logistic and Cox regression models adds consistency to the observed associations.

At the same time, several limitations must be acknowledged. The retrospective design inherently limits causal inference and leaves room for residual confounding despite multivariable adjustment. Although classical oncologic predictors were accounted for, unmeasured perioperative or biological variables may have contributed to the observed associations. Although selected perioperative metabolic parameters, including glycemic variation, intraoperative lactate dynamics, and IL-6 response, were available and incorporated into the analysis, comprehensive metabolomic profiling was not performed. More detailed characterization of metabolic pathways, such as insulin resistance indices, broader cytokine panels, or high-resolution metabolomic signatures, could provide deeper mechanistic insight into the interaction between perioperative systemic modulation and tumor biology. Therefore, the metabolic interpretation of our findings remains inferential rather than pathway-specific.

Furthermore, IL-6 is a pleiotropic cytokine strongly influenced by surgical trauma and perioperative inflammatory activation. Consequently, changes in IL-6 levels should be interpreted primarily as markers of systemic stress response rather than tumor-specific biological activity.

Furthermore, early postoperative PSA dynamics, although clinically meaningful, represent a surrogate endpoint. While early biochemical suppression is associated with favorable oncologic prognosis, it cannot substitute for long-term recurrence-free, metastasis-free, or overall survival outcomes. Longer follow-up is required to determine whether the observed differences in early PSA behavior translate into sustained oncologic benefit. An additional important limitation relates to the non-randomized nature of perioperative lidocaine administration. The decision to use intravenous lidocaine was based on the attending anesthesiologist’s clinical practice and institutional protocol during the study period rather than random allocation. Consequently, although baseline characteristics and major surgical parameters were well balanced between groups, unmeasured differences in perioperative anesthetic management may still have been present. Variables such as intraoperative opioid dosing, fluid strategies, vasopressor administration, and other anesthetic factors could potentially influence both metabolic stress responses and early postoperative recovery. In addition, detailed stratification of nerve-sparing technique was not consistently available in the retrospective dataset and therefore could not be included as a covariate in the regression models. However, all procedures were performed by the same experienced robotic surgical team using a standardized surgical approach, which likely minimized major variability related to surgical technique.

While our multivariable models adjusted for major oncologic determinants, residual confounding related to anesthetic management cannot be completely excluded. Therefore, the observed associations should be interpreted cautiously as hypothesis-generating rather than as evidence of a causal biological effect.

An additional methodological consideration relates to the analysis of time to first detectable PSA. In routine clinical practice, PSA measurements are obtained at predefined follow-up visits rather than through continuous monitoring. Consequently, the exact timing of PSA detectability is interval-censored between scheduled assessments. Although Cox proportional hazards models are commonly used in similar clinical studies evaluating time-to-event outcomes with periodic follow-up, this approach assumes an approximate event time based on the first recorded detectable value. Therefore, the results of the time-to-event analysis should be interpreted with caution, acknowledging that the discrete nature of PSA assessments may introduce a degree of temporal imprecision. Perioperative opioid exposure and anesthetic depth monitoring variables were not incorporated into the regression models and may represent additional factors influencing perioperative stress responses and metabolic biomarkers.

Accordingly, the Cox proportional hazards analysis should be interpreted primarily as an exploratory assessment of early postoperative PSA detectability rather than as a precise estimate of event timing. Importantly, the outcomes evaluated in the present study represent early postoperative PSA dynamics rather than established biochemical recurrence. Therefore, the findings should not be interpreted as evidence of an effect on long-term oncologic outcomes.

Future investigations should ideally include prospective designs with predefined perioperative metabolic and immune profiling. The integration of more detailed biomarkers, such as cytokine panels, metabolic signatures, or immune cell functional assays, could provide deeper mechanistic insight into the interaction between perioperative systemic modulation and tumor biology. Additionally, exploring whether perioperative metabolic stabilization strategies influence long-term oncologic outcomes would help clarify the clinical relevance of the perioperative metabolic window. A more refined understanding of these interactions may ultimately contribute to integrated surgical-anesthetic strategies that consider not only technical tumor.

## 5. Conclusions

In this retrospective observational cohort of patients undergoing robotic-assisted radical prostatectomy, perioperative intravenous lidocaine administration was independently associated with a higher likelihood of achieving undetectable PSA during early postoperative follow-up. This association was observed at 6–12 weeks, 3 months, and 6 months and remained significant after adjustment for preoperative PSA, pathological stage, ISUP grade group, and surgical margin status.

Patients receiving lidocaine also demonstrated greater intraoperative hemodynamic stability, fewer hypotensive and hypertensive episodes, reduced vasopressor requirements, shorter time to extubation, and reduced length of hospital stay. Although differences in inflammatory markers did not reach statistical significance, a consistent directional trend toward lower postoperative inflammatory activation was observed in the lidocaine group. The attenuation of perioperative glycemic elevation, lactate dynamics, and IL-6 response observed in the lidocaine group further supports the concept that perioperative metabolic–inflammatory modulation during the perioperative window may represent a potentially modifiable dimension associated with early postoperative PSA dynamics.

Classical pathological factors remained the dominant predictors of early biochemical outcomes, as expected. However, the persistence of the lidocaine association after multivariable adjustment suggests that perioperative systemic conditions may contribute to early PSA dynamics alongside established tumor-related determinants.

These findings suggest a potential association between perioperative lidocaine administration, attenuation of perioperative metabolic-inflammatory activation, and early PSA dynamics following robotic-assisted radical prostatectomy. However, given the retrospective and non-randomized nature of the study and the possibility of residual perioperative confounding, these observations should be interpreted as exploratory and hypothesis-generating. Prospective randomized studies incorporating standardized anesthetic protocols and comprehensive perioperative biomarker profiling will be necessary to determine whether perioperative systemic modulation truly influences early oncologic biomarkers.

## Figures and Tables

**Table 1:** Baseline demographic, clinical, and pathological characteristics of the study population.

Parameter	Lidocaine Group (*n* = 90)	Control Group (*n* = 90)	*p*-Value
Age (years) *	64.3 ± 6.8	65.1 ± 7.1	0.49
BMI (kg/m^2^) *	27.4 ± 2.9	27.9 ± 3.0	0.38
ASA physical status (I/II/III) ***	14/56/20	12/58/20	0.87
Preoperative PSA (ng/mL) **	8.0 (5.5–11.8)	8.4 (5.8–12.3)	0.58
Preoperative leukocytes (×10^9^/L) *	7.3 ± 1.9	7.5 ± 1.8	0.61
Preoperative NLR *	2.4 ± 0.9	2.6 ± 1.0	0.47
Preoperative CRP (mg/L) *	3.3 ± 2.2	3.5 ± 2.4	0.65
Clinical stage (≤T2/≥T3) ***	67/23	65/25	0.74
Biopsy ISUP grade group (1/2/3–5) ***	23/38/29	22/40/28	0.95
Pathological stage (pT2/pT3a-b) ***	59/31	57/33	0.76
Final ISUP grade group (1–2/3–5) ***	49/41	47/43	0.82
Positive surgical margins ***	18 (20.0%)	21 (23.3%)	0.59
Pelvic lymph node dissection performed, *n* (%) ***	41 (45.6%)	43 (47.8%)	0.77
Pathological lymph node involvement (pN+) ***	5 (5.6%)	7 (7.8%)	0.55
Operative time (minutes) *	169 ± 25	172 ± 26	0.44
Estimated blood loss (mL) *	230 ± 75	245 ± 85	0.29

* Data are presented as mean ± standard deviation; ** Data are presented as median (interquartile range); *** Data are presented as number (percentage). Between-group comparisons were performed using the independent samples *t*-test for normally distributed continuous variables, the Mann–Whitney U test for non-normally distributed variables, and the Chi-square or Fisher’s exact test for categorical variables. Abbreviations: BMI, body mass index; ASA, American Society of Anesthesiologists; PSA, prostate-specific antigen; NLR, neutrophil-to-lymphocyte ratio; CRP, C-reactive protein; ISUP, International Society of Urological Pathology.

**Table 2:** Surgical and intraoperative characteristics.

Parameter	Lidocaine Group (*n* = 90)	Control Group (*n* = 90)	*p*-Value
Duration of surgery (min) *	170 ± 26	173 ± 28	0.45
Duration of anesthesia (min) *	205 ± 30	209 ± 32	0.39
Pneumoperitoneum pressure (mmHg) *	12.5 ± 1.2	12.6 ± 1.3	0.62
Time in Trendelenburg (min) *	145 ± 22	148 ± 25	0.41
Estimated blood loss (mL) *	230 ± 75	245 ± 85	0.29
Fluids administered (mL) *	2100 ± 450	2200 ± 480	0.18
Blood transfusion, *n* (%) **	4 (4.4%)	6 (6.7%)	0.51
Urine output (mL) *	520 ± 160	505 ± 170	0.57
Type of procedure **			
- RARP only	49 (54.4%)	47 (52.2%)	0.77
- RARP + pelvic lymph node dissection	41 (45.6%)	43 (47.8%)	0.77

* Data are presented as mean ± standard deviation; ** Data are presented as number (percentage). Between-group comparisons were performed using the independent samples *t*-test for normally distributed continuous variables and the Chi-square or Fisher’s exact test for categorical variables. Abbreviations: RARP, robotic-assisted radical prostatectomy.

**Table 3:** Intraoperative hemodynamic parameters.

Parameter	Lidocaine Group (*n* = 90)	Control Group (*n* = 90)	*p*-Value
Mean Arterial Pressure (MAP, mmHg) *			
T0	94 ± 11	95 ± 12	0.68
T1	82 ± 10	78 ± 12	**0.03**
T2	88 ± 9	92 ± 11	**0.02**
T3	90 ± 8	94 ± 10	**0.01**
T4	89 ± 9	93 ± 11	**0.02**
T5	92 ± 10	95 ± 12	0.08
Heart Rate (beats per minute) *			
T0	72 ± 9	73 ± 10	0.59
T1	68 ± 8	72 ± 9	**0.01**
T2	70 ± 9	76 ± 10	**0.002**
T3	71 ± 8	77 ± 9	**<0.001**
T4	72 ± 9	78 ± 10	**0.001**
T5	74 ± 10	79 ± 11	**0.01**
Hemodynamic events and interventions			
Episodes of MAP <65 mmHg (*n* per patient) *	0.6 ± 0.9	1.2 ± 1.4	**0.004**
Duration of hypotension (min) *	4.8 ± 6.2	9.6 ± 10.8	**0.002**
Episodes of hypertension (SBP > 160 mmHg) *	0.7 ± 1.0	1.4 ± 1.6	**0.006**
Vasopressor use, *n* (%) **	24 (26.7%)	39 (43.3%)	**0.02**
Number of vasopressor boluses *	1.1 ± 1.5	2.0 ± 2.4	**0.01**

* Data are presented as mean ± standard deviation; ** Data are presented as numbers (percentage). T0: baseline before induction; T1: after induction; T2: after pneumoperitoneum; T3: after Trendelenburg positioning; T4: 60 min intraoperatively; T5: end of surgery. Between-group comparisons were performed using independent samples *t*-test for continuous variables and Chi-square test for categorical variables. Bold: Statistically significant at *p* < 0.05.

**Table 4:** Intraoperative physiological and metabolic parameters.

Parameter	Lidocaine Group (*n* = 90)	Control Group (*n* = 90)	*p*-Value
End-Tidal CO_2_ (EtCO_2_, mmHg)			
T0	36.2 ± 3.1	36.4 ± 3.2	0.72
T1	35.0 ± 3.0	35.2 ± 3.1	0.66
T2	39.1 ± 3.6	39.6 ± 3.7	0.40
T3	40.2 ± 3.8	40.8 ± 3.9	0.34
T4	40.0 ± 3.7	40.7 ± 3.8	0.22
T5	37.1 ± 3.3	37.5 ± 3.4	0.45
Arterial PaCO_2_ (mmHg)			
T0	39.0 ± 3.6	39.2 ± 3.7	0.74
T1	38.2 ± 3.5	38.5 ± 3.6	0.60
T2	43.1 ± 4.1	43.7 ± 4.3	0.39
T3	44.2 ± 4.3	45.0 ± 4.5	0.28
T4	44.0 ± 4.2	44.8 ± 4.4	0.27
T5	40.1 ± 3.8	40.6 ± 3.9	0.44
Peak Airway Pressure (Ppeak, cmH_2_O)			
T0	18.1 ± 3.0	18.3 ± 3.1	0.69
T3	26.8 ± 4.1	27.4 ± 4.3	0.36
T4	26.2 ± 4.0	26.9 ± 4.2	0.29
Plateau Pressure (Pplat, cmH_2_O)			
T0	13.6 ± 2.4	13.8 ± 2.5	0.58
T3	19.6 ± 3.2	20.1 ± 3.3	0.34
T4	19.3 ± 3.1	19.9 ± 3.2	0.25
Dynamic Compliance (mL/cmH_2_O)			
T0	41.5 ± 8.0	41.0 ± 8.2	0.73
T3	30.4 ± 6.6	29.6 ± 6.8	0.46
T4	31.1 ± 6.8	30.2 ± 7.0	0.42
Minute Ventilation (L/min)			
T0	7.3 ± 1.1	7.4 ± 1.1	0.64
T3	8.8 ± 1.3	9.0 ± 1.4	0.39
T4	8.7 ± 1.3	8.9 ± 1.4	0.41
SpO_2_ (%)			
T0	98.6 ± 0.9	98.5 ± 1.0	0.52
T3	97.9 ± 1.2	97.8 ± 1.3	0.65
T4	98.0 ± 1.1	97.9 ± 1.2	0.63
*Blood Glucose*			
T0	94 ± 12	95 ± 11	0.71
T4	118 ± 18	132 ± 22	0.002
24 h post-op	124 ± 20	142 ± 26	0.001
Δ *Glucose (T4* − *T0)*	+24 ± 15	+37 ± 18	0.004
*Arterial Lactate* (mmol/L)			
Baseline	1.2 ± 0.3	1.2 ± 0.4	0.88
Peak intraoperative	1.6 ± 0.5	2.2 ± 0.7	0.004
Δ *Lactate*	+0.4 ± 0.3	+1.0 ± 0.5	<0.01
*IL6* (pg/mL)			
Baseline	3.5 ± 1.8	3.6 ± 2.0	0.82
6–24 h postoperator (peak)	58 ± 24	82 ± 35	0.001
Δ *IL-6* (*post -baseline*)	+54	+78	<0.01

Data are presented as mean ± standard deviation. T0: baseline before induction; T1: after induction; T2: after pneumoperitoneum; T3: after Trendelenburg positioning; T4: 60 min intraoperatively; T5: end of surgery. Between-group comparisons were performed using the independent samples *t*-test. Abbreviations: EtCO_2_, end-tidal carbon dioxide; PaCO_2_, arterial partial pressure of carbon dioxide; Ppeak, peak inspiratory pressure; Pplat, plateau pressure; SpO_2_, peripheral oxygen saturation. Metabolic variables central to the study hypothesis (blood glucose, Δ glucose, arterial lactate, Δ lactate, and IL-6 parameters) are presented in italics to facilitate visual identification of indicators related to perioperative metabolic–inflammatory modulation.

**Table 5:** Postoperative outcomes.

Outcome	Lidocaine Group (*n* = 90)	Control Group (*n* = 90)	*p*-Value
Time to extubation (min) *	9.5 ± 4.2	12.1 ± 5.3	0.001
PONV, *n* (%) **	11 (12.2%)	20 (22.2%)	0.08
Postoperative ileus, *n* (%) **	5 (5.6%)	12 (13.3%)	0.07
Length of hospital stay (days) ***	4 (3–5)	5 (4–6)	0.01
Overall complications (Clavien-Dindo ≥ II) **	13 (14.4%)	21 (23.3%)	0.13
Clavien-Dindo Grade I **	8 (8.9%)	12 (13.3%)	0.34
Clavien-Dindo Grade II **	11 (12.2%)	16 (17.8%)	0.29
Clavien-Dindo Grade III-IV **	3 (3.3%)	6 (6.7%)	0.31
Readmission within 30 days **	6 (6.7%)	11 (12.2%)	0.19

* Data are presented as mean ± standard deviation; ** Data are presented as number (percentage); *** Data are presented as median (interquartile range). Between-group comparisons were performed using the independent samples *t*-test, Chi-square/Fisher’s exact test, or Mann–Whitney U test, as appropriate.

**Table 6:** Multivariable logistic regression models for undetectable PSA at different time points after RARP.

Variable	6–12 Weeks OR (95% CI)	*p*-Value	3 Months OR (95% CI)	*p*-Value	6 Months OR (95% CI)	*p*-Value
Perioperative IV lidocaine (yes vs. no)	2.10 (1.15–3.85)	0.016 *	1.95 (1.05–3.64)	0.034 *	2.05 (1.08–3.90)	0.028 *
Preoperative PSA (per 1 ng/mL increase)	0.90 (0.84–0.97)	0.006 *	0.89 (0.83–0.96)	0.003 *	0.88 (0.81–0.95)	0.002 *
Pathological stage (pT3 vs. pT2)	0.46 (0.25–0.86)	0.015 *	0.44 (0.23–0.84)	0.012 *	0.41 (0.21–0.81)	0.010 *
Final ISUP grade group (3–5 vs. 1–2)	0.53 (0.29–0.96)	0.037 *	0.50 (0.27–0.92)	0.026 *	0.48 (0.25–0.91)	0.024 *
Positive surgical margins (yes vs. no)	0.48 (0.24–0.96)	0.038 *	0.44 (0.22–0.90)	0.024 *	0.42 (0.20–0.88)	0.021 *

Undetectable PSA (<0.1 ng/mL) at each time point (Yes/No). Separate multivariable logistic regression analyses were performed for each time point. ORs are adjusted for all variables shown. * Statistically significant at *p* < 0.05.

**Table 7:** Sensitivity multivariable logistic regression model including perioperative metabolic parameters (Δ IL-6 and Δ lactate) for undetectable PSA (<0.1 ng/mL) at 6–12 weeks after RARP.

Variable	Adjusted OR (95% CI)	*p*-Value
Perioperative IV lidocaine (yes vs. no)	1.78 (1.01–3.30)	0.046
Preoperative PSA (per 1 ng/mL increase)	0.90 (0.84–0.97)	0.006
Pathological stage (pT3 vs. pT2)	0.48 (0.25–0.92)	0.027
Final ISUP grade group (3–5 vs. 1–2)	0.55 (0.30–0.99)	0.048
Positive surgical margins (yes vs. no)	0.50 (0.25–0.98)	0.043
Δ IL-6 (per 10 pg/mL increase)	0.92 (0.86–0.99)	0.024
Δ Lactate (per 0.5 mmol/L increase)	0.83 (0.69–1.00)	0.052

Outcome = undetectable PSA (<0.1 ng/mL) at 6–12 weeks (Yes/No). ORs are adjusted for all variables shown. Δ IL-6 = postoperative (6–24 h) minus baseline; Δ lactate = peak intraoperative minus baseline. Continuous metabolic variables were scaled for clinical interpretability (per 10 pg/mL and per 0.5 mmol/L, respectively).

**Table 8:** Multivariable Cox proportional hazards regression for time to first early postoperative detectable PSA (≥0.1 ng/mL) after RARP.

Variable	HR	95% CI	*p*-Value
Perioperative IV lidocaine (yes vs. no)	0.58	0.37–0.92	0.021 *
Preoperative PSA (per 1 ng/mL increase)	1.10	1.05–1.16	<0.001 *
Pathological stage (pT3 vs. pT2)	2.05	1.30–3.22	0.002 *
Final ISUP grade group (3–5 vs. 1–2)	1.78	1.13–2.80	0.013 *
Positive surgical margins (yes vs. no)	1.90	1.18–3.05	0.008 *

* HR < 1 indicates lower hazard (lower risk) of developing detectable PSA. Detectable PSA refers to early postoperative PSA detectability (≥0.1 ng/mL) and should not be interpreted as biochemical recurrence.

## Data Availability

The data supporting the findings of this study are available from the corresponding author upon reasonable request. Due to institutional data protection policies of the participating hospital, the datasets are not publicly available.
